# Knowledge domain and emerging trends in HIV pre-exposure prophylaxis: A visualization analysis *via* CiteSpace

**DOI:** 10.3389/fmicb.2023.1099132

**Published:** 2023-03-16

**Authors:** Xi Chen, Yu Lai

**Affiliations:** School of Basic Medicine, Chengdu University of Traditional Chinese Medicine, Chengdu, China

**Keywords:** HIV/AIDS, pre-exposure prophylaxis, CiteSpace, visualization analysis, emerging trends

## Abstract

**Background:**

As an effective strategy that reduces transmission among people at high risk of human immunodeficiency virus (HIV), pre-exposure prophylaxis (PrEP) has revolutionized HIV prevention. Our study aims to provide a reference for the development of relevant research and the formulation of prevention and control policies for HIV.

**Methods:**

Through CiteSpace software, this study aims to present a comprehensive overview of the HIV PrEP knowledge structure, hotspots, and frontiers. We searched the Web of Science Core Collection for studies published between 2012 and 2022 related to HIV PrEP, and 3,243 papers remained after selection.

**Results:**

The number of HIV PrEP publications has increased over the past few years. A close integration and exchange of HIV PrEP research findings has taken place between countries and authors. Major ongoing research trends include long-term injection PrEP, the impact of chlamydia on HIV PrEP, and individual awareness of and attitudes toward HIV PrEP. Thus, more attention should be paid to innovations and breakthroughs in drugs, the factors that affect HIV transmission and susceptibility, and the future promotion of public acceptance of HIV PrEP.

**Conclusion:**

This study offers a systematic, objective, and comprehensive analysis of the related articles. It will assist scholars in understanding the dynamic evolution of HIV PrEP research and identifying future research areas to better advance the development of the field.

## 1. Introduction

Since it was first reported in 1981, human immunodeficiency virus (HIV)/acquired immune deficiency syndrome (AIDS) has evolved into one of the greatest health challenges worldwide (De Cock et al., 2012). AIDS has already claimed the lives of 36.3 million people around the world, and approximately 38 million people live with HIV (WHO, 2022). Globally, there were an estimated 680,000 AIDS-related deaths and 1.5 million new infections in 2020 (WHO, 2022). At present, curing HIV infection, meaning completely eradicating the virus from the patient, remains a difficult goal to achieve. In this situation, stemming the global epidemic by preventing further infections from the virus is particularly important. Developing a safe and effective HIV-1 vaccine is undoubtedly the best solution. Unfortunately, due to the genetic diversity and mutability of HIV-1 and the virus’s capacity to evade adaptive immune responses, this goal has been elusive.

Thus, HIV PrEP, which refers to the prevention of HIV infection through antiretroviral treatment before exposure, is a critical measure (McCormack et al., 2016). PrEP for HIV was approved by the FDA in 2012 (Petroll et al., 2017). With the promotion of the use of emtricitabine/tenofovir (Truvada) for people with HIV-negative status, a new era of HIV prevention began (Petroll et al., 2017; Glidden, 2019). Tenofovir is a reverse transcriptase (RT) inhibitor. Antiviral agents can prevent viral single-stranded genomic RNA from being converted into double-stranded DNA (dsDNA), which is necessary for integration into the host genome. Several drugs have been approved for inhibiting RT DNA polymerase activity, including nucleoside analogs (nucleoside RT inhibitors (NRTIs)) and non-nucleoside RT inhibitors (NNRTIs). This class of drugs includes tenofovir, an acyclic analog of monophosphate deoxyadenosine. Before being incorporated into the DNA of the provirus, NRTIs as prodrugs must be converted to triphosphorylated derivatives. In approved NRTIs, the ribose ring lacks a 3’ OH, which acts as a chain terminator, preventing DNA synthesis (Menendez-Arias and Delgado, 2022). Studies have shown that consistent (at least 4 times per week) use of PrEP can decrease the risk of HIV sexual transmission by 99% and injection drug use transmission by at least 74% (Choopanya et al., 2013; Liu et al., 2016; McCormack et al., 2016; Marcus et al., 2017). Increasing evidence suggests that due to the high effectiveness of HIV PrEP drugs and the harmfulness of HIV, it is necessary to quantitatively analyze the research status, hotspots, and frontiers of HIV PrEP.

Bibliometrics uses statistical analysis of published data to evaluate and track the development of particular fields (Xu et al., 2022). It is heavily reliant on technologies for visual processing, such as CiteSpace. In recent years, CiteSpace has been widely used in various fields, but a comprehensive evaluation and summary of HIV PrEP literature characteristics, research directions, and depth are lacking. Therefore, the research trends in HIV PrEP must be summarized and analyzed to provide directions and a basis for future studies. We aimed to provide a comprehensive understanding of the developments in the research on HIV PrEP by analyzing the remarkable achievements in the past 20 years. Through CiteSpace, we concentrate on the network of authors, countries, and co-occurrence keywords, journal analysis, keyword bursts, cocited reference analysis, and cluster analysis and discuss the hotspots and trends of HIV PrEP.

## 2. Materials and methods

### 2.1. Data collection and processing

Web of Science (WOS) is one of the most commonly used academic database sources, with over 12,000 significant publications (Wu et al., 2021). It is largely acknowledged as the most complete and credible resource for bibliometric analysis when compared to other databases such as Scopus and PubMed. To guarantee that the original data are comprehensive, accurate, and highly reliable, we selected the literature through the Science Citation Index—Expanded version (SCIE) of the Web of Science Core Collection (WoSCC). The WoSCC from Clarivate Analytics is one of the databases that provide comprehensive research types and timely data updates. It is widely used for bibliometric analysis. The retrieved dataset is a dataset about “HIV PrEP” for the period from 2012 to 2022. Specifically, the data retrieval strategies were set as follows:

#1: TS = (“Pre-Exposure Prophylaxi*”) OR TS = PrEP.

#2: TS = HIV OR TS = (“Human Immunodeficiency Viru*”) OR TS = (“AIDS Viru*”) OR TS = HTLV-III.

#1 AND #2.

Inclusion Criteria: Only articles and reviews were concluded based on the document type. The only available language is English. Both researchers independently assessed the publication titles and abstracts and eliminated studies that had no connection to HIV PrEP.

The selected literature covers a broad range and has strong representativeness. Moreover, the data were all acquired on October 31, 2022, to minimize bias because of database updates. After the duplicate articles were removed, 3,243 valid studies remained. The image-based screening process and the data used for the study can be viewed in the Supplemental materials. The data were exported from the WoSCC, and “full record and cited references” were converted into plain text format. CiteSpace software, Version 5.8.R3, was employed for the following analysis. We mapped the visualization knowledge figures using the main software functions, such as time slicing, thresholding, modeling, pruning, merging, and mapping.

### 2.2. CiteSpace software analysis

CiteSpace, used in this study, is a Java-based application that performs the bibliometric and visual analysis. This software was created by Chen in early 2004 (Chen, 2004). The major input source for CiteSpace is the Web of Science (WOS). The procedural steps are time slicing, thresholding, modeling, pruning, merging, and mapping. These process steps and other CiteSpace software-related knowledge are explained in a book by Chaomei Chen (Li and Chen, 2022). The software can be used to visualize trends and models in scientific literature over a specific period of time (Zhong et al., 2020). In CiteSpace, one can perform coreference, coauthor, and co-occurrence keyword analysis, making it easier to explore a research area. The software can visualize knowledge graphs consisting of nodes and links. Each node in a graph represents an element to be analyzed. The lines connecting nodes are considered co-occurrence or cocitation relationships. In addition, nodes with high centrality (> 0.1) are thought of as turning points or key points in the field. The greater the centrality, the higher the representation of the corresponding research contents in a certain period of time. A CiteSpace visual knowledge map can help identify the nature of a research frontier, label a specialty, and detect emerging trends and sudden changes in a field.

The parameters of CiteSpace were set as below:

This study set the time span to 2012–2022, and the length per time slice to “1.” In coauthorship analysis, the selection criteria are the top 30 and pruning to the minimum spanning tree (MST). In countries analysis, the top 30 most frequently occurring items were chosen. In cocited references analysis and keyword analysis, the top 20% of most cited or occurring items were selected, and a pruning algorithm was adopted.

## 3. Results

### 3.1. Publication year analysis

**Figure 1:**
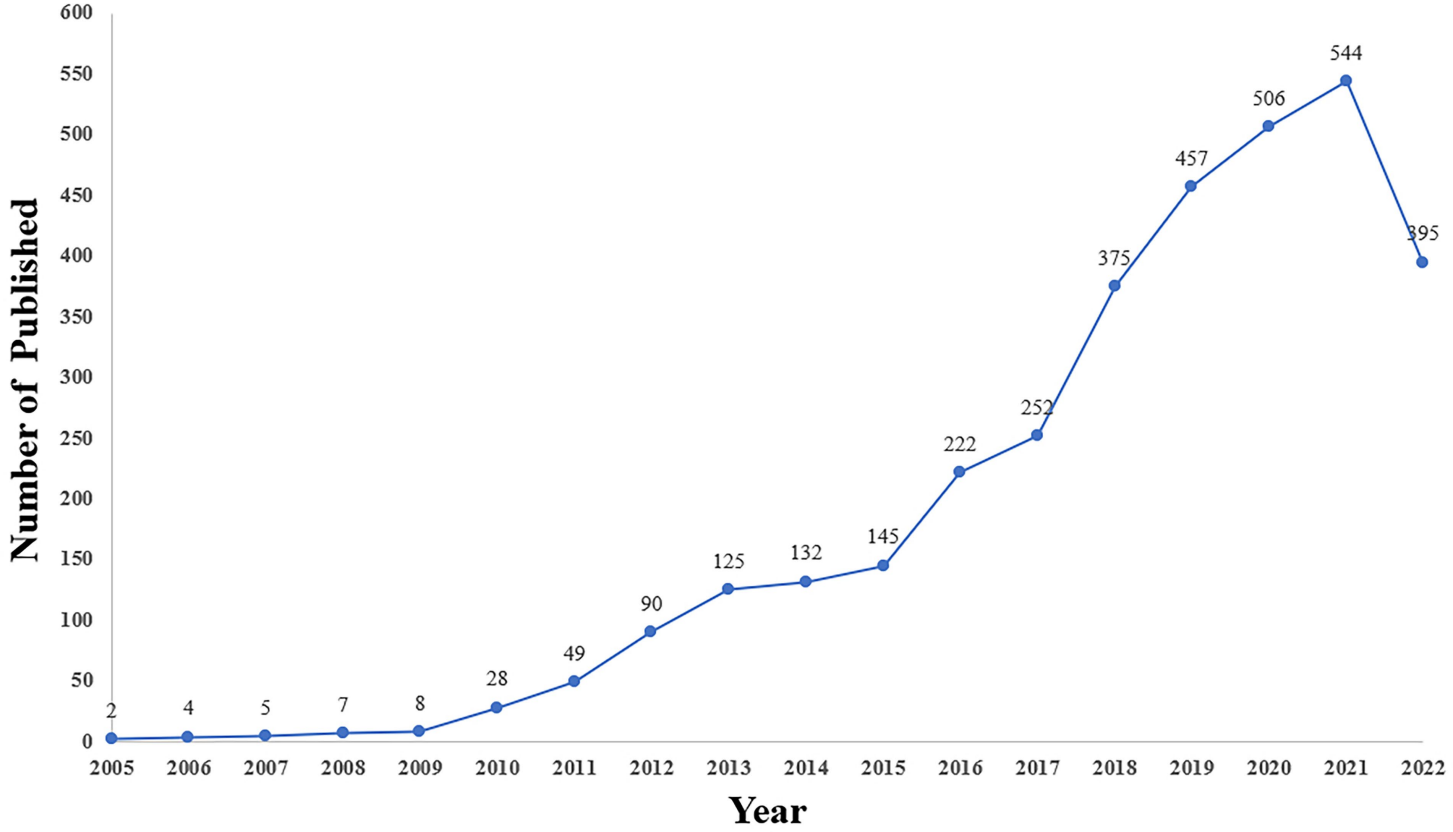
Annual trend chart of publications.

Academic paper publications can reflect changes in a discipline’s level of development. As evidenced in Figure 1, HIV PrEP was not initially recognized by the medical community. There were only a few related articles. And since 2010, there has been a growing interest in HIV PrEP, with the number of published increasing from 28 in 2010 to 544 in 2021. The number of articles publications on the topic peaked in 2021. Overall, these findings indicate that HIV PrEP has emerged as a research hotspot, capturing global scientific attention.

### 3.2. Coauthorship analysis

**Figure 2:**
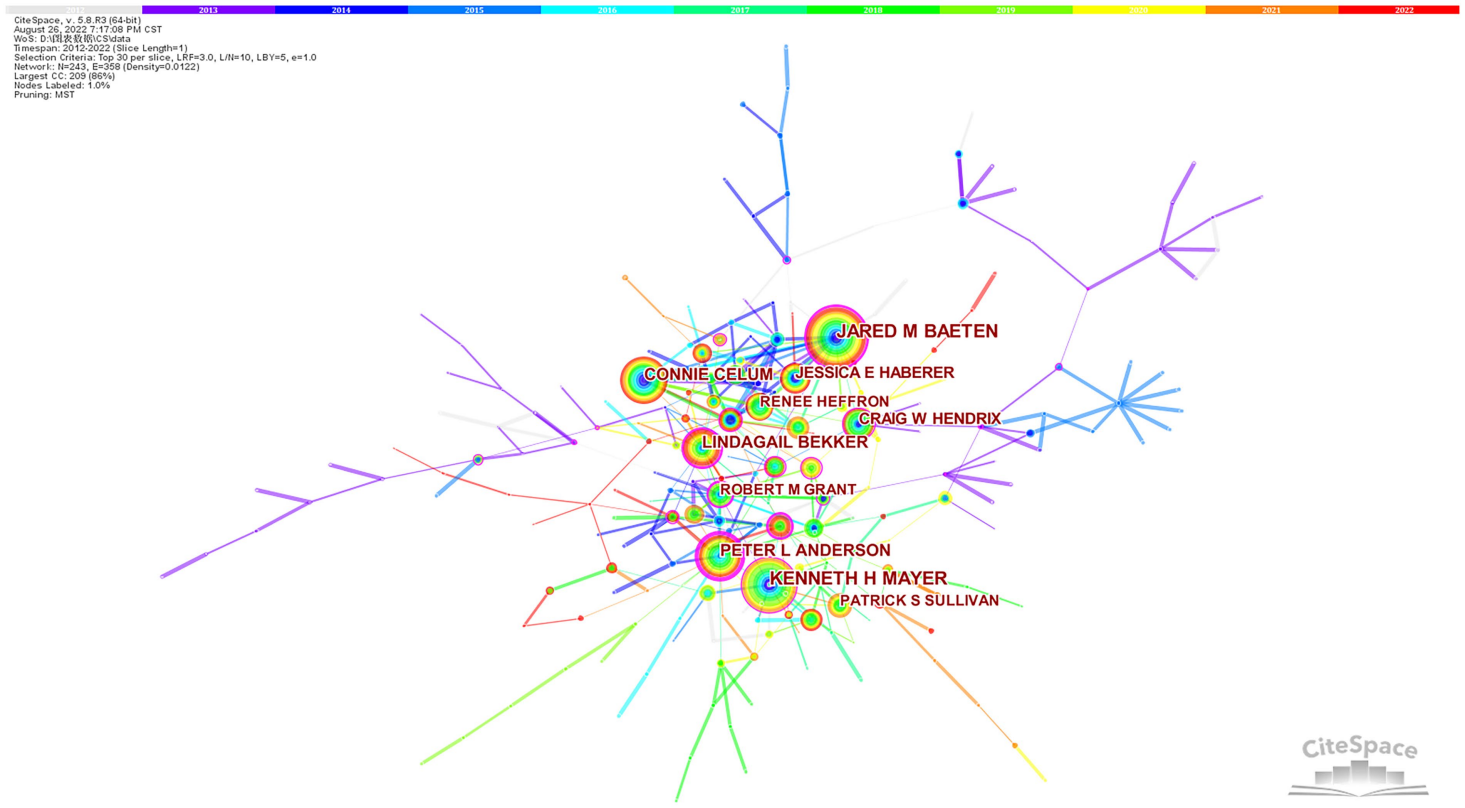
The network of coauthors.

To discover high-influence authors in HIV PrEP, we used CiteSpace to present the coauthor network. We generated a distribution map (Figure 2) of coauthors with 243 nodes and 358 connections. The size of a circle represents the number of studies published by the author. Table 1 lists the top 10 core authors with a large number of publications. The most productive author in HIV PrEP research was JARED BAETEN, with 154 articles, followed by KENNETH MAYER (121), CONNIE CELUM (87), PETER ANDERSON (81), and LINDAGAIL BEKKER (74). In the diagram, links between different authors represent collaboration. The thickness of the lines represents the strength of the cooperation, and the thicker the line is, the more collaboration between individual authors. As shown in Figure 2, there were several important groups of authors, each of which usually contained two or more core authors. This is evident from the fact that many authors worked with a relatively stable group of collaborators.

**Table 1:** The top 10 most productive authors.

Rank	Author	Count of articles	Year of first articles
1	JARED M BAETEN	154	2012
2	KENNETH H MAYER	121	2012
3	CONNIE CELUM	87	2012
4	PETER L ANDERSON	81	2014
5	LINDAGAIL BEKKER	74	2012
6	CRAIG W HENDRIX	56	2013
7	JESSICA E HABERER	51	2012
8	PATRICK S SULLIVAN	48	2014
9	ROBERT M GRANT	47	2012
10	RENEE HEFFRON	46	2014

### 3.3. Country analysis

**Figure 3:**
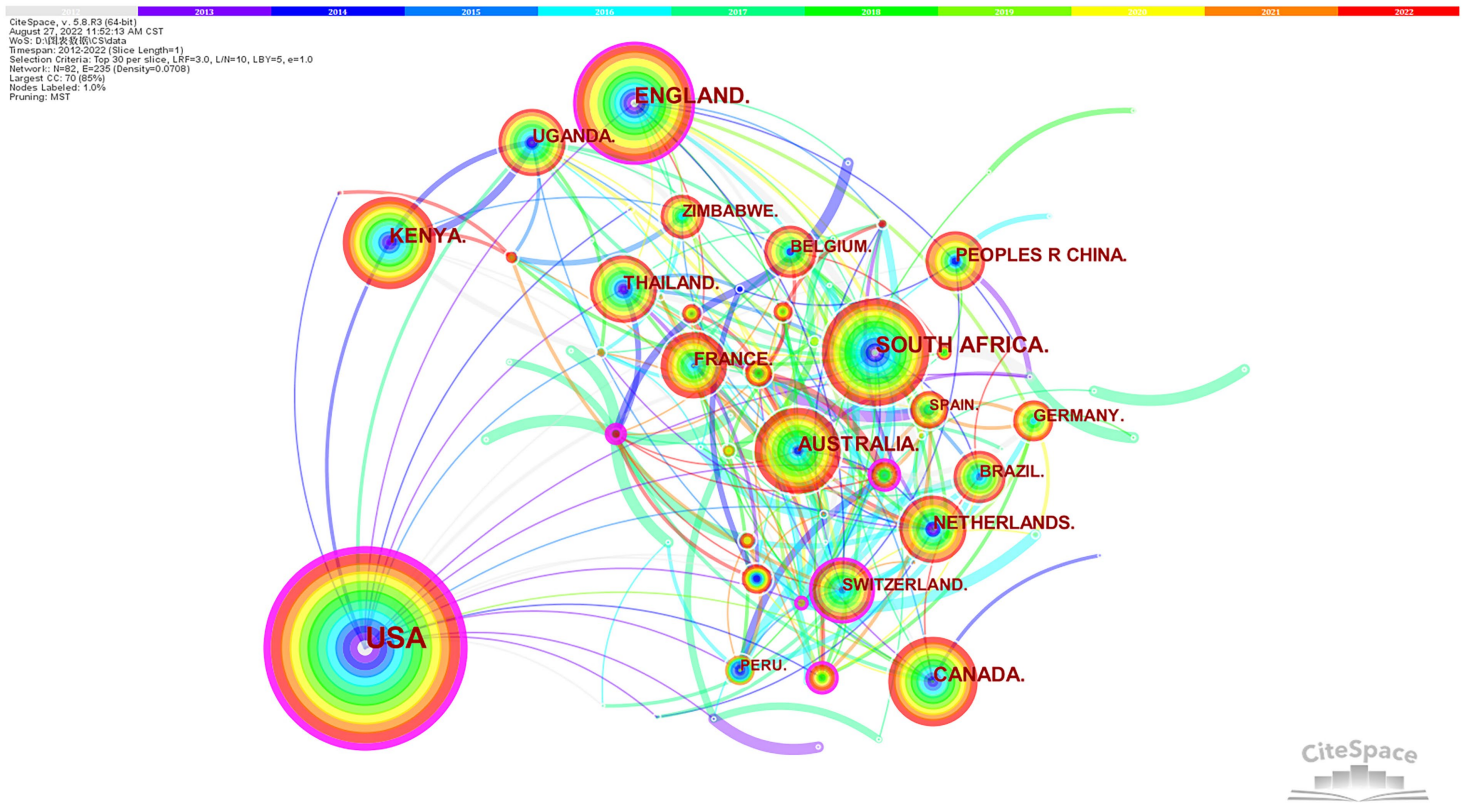
The network of countries.

Figure 3 displays the cocountry network in HIV PrEP research. The size of the circle represents the number of publications in the country. The total publications of Table 2 show that the USA has published 2,270 (70%) papers, making a significant contribution to the related research. After the USA, the top five countries are the UK, South Africa, Kenya, and Australia. Furthermore, the United Based on the multiple lines in the graph, we can conclude that countries collaborated actively.

**Table 2:** The top 10 counties.

Rank	Country	Count
1	USA	2,270
2	ENGLAND	380
3	SOUTH AFRICA	329
4	KENYA	205
5	AUSTRALIA	196
6	CANADA	192
7	FRANCE	138
8	UGANDA	132
9	NETHERLANDS	112
10	PEOPLES R CHINA	112

### 3.4. Journal analysis

**Figure 4:**
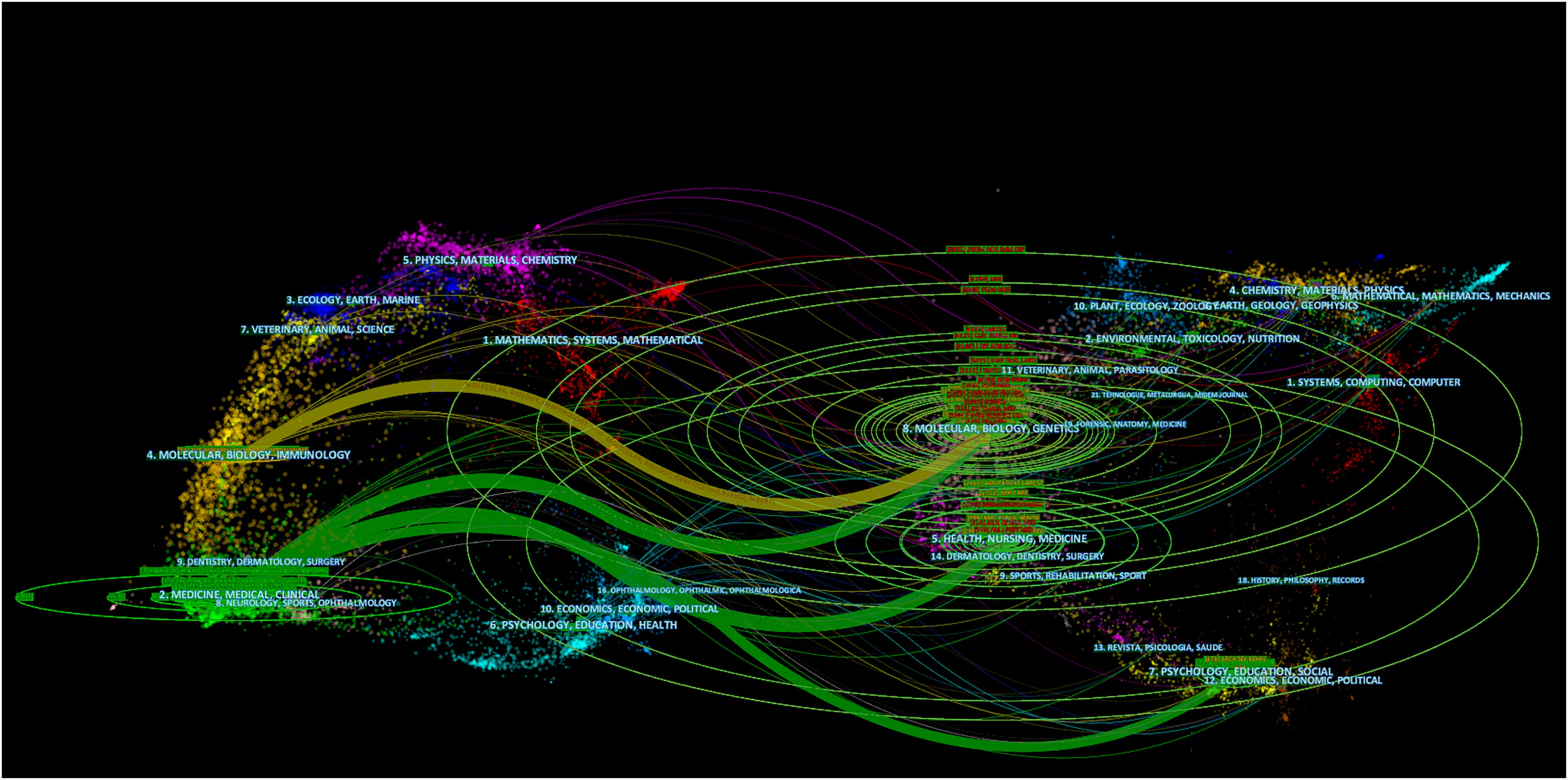
The dual-map overlay of journals.

A dual-map overlay of journals is shown in Figure 4, with citing journals on the left and cited journals on the right. The colored paths indicate the relationships between them. The map reveals that studies published in Medicine/Medical/Clinical journals usually cite studies published in Molecular/Biology/Genetics and Health/Nursing/Medicine journals.

### 3.5. Cocited references and cluster analysis

**Figure 5:**
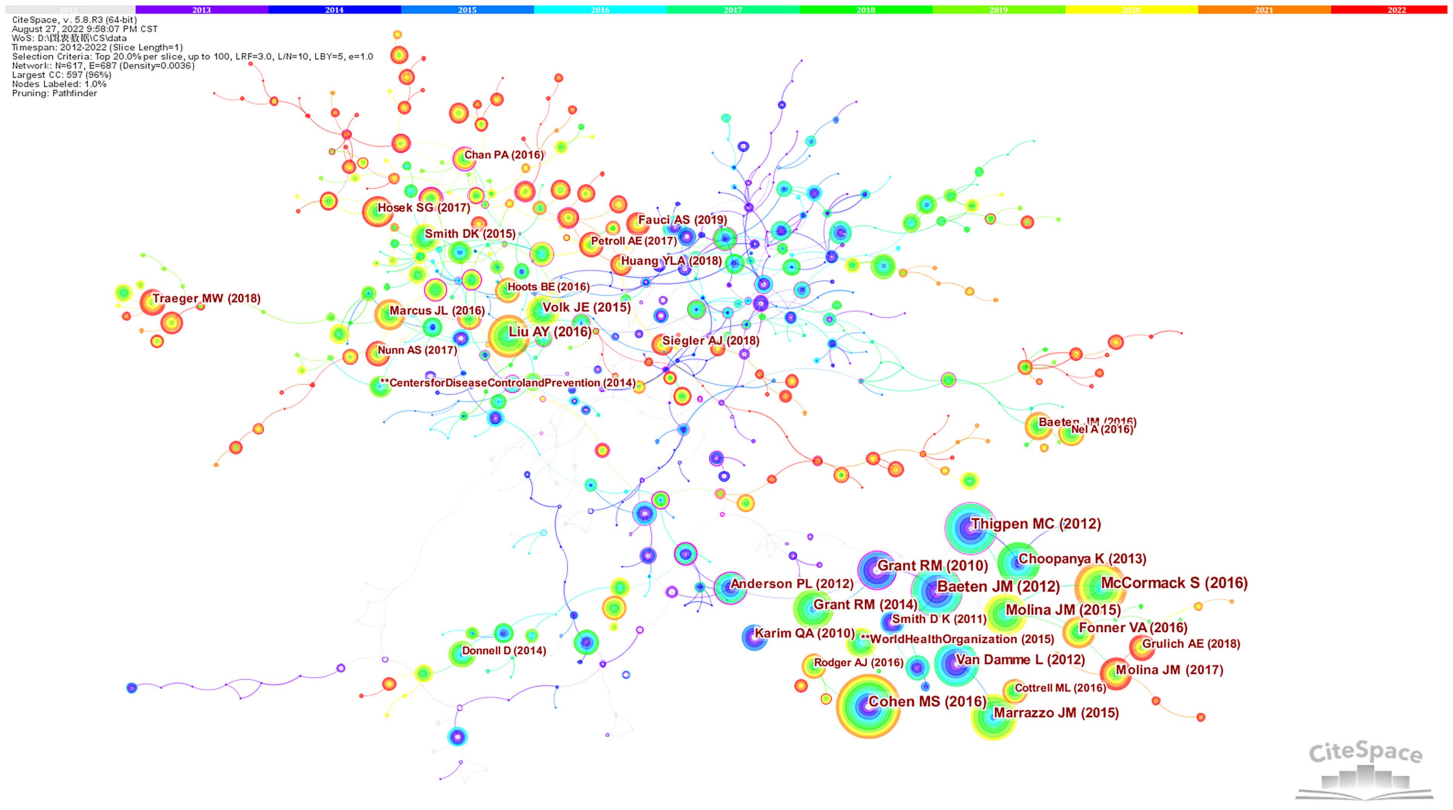
Reference cocitation map.

Figure 5 shows an article coreference system. As a result, 617 unique nodes and 1,196 links were generated. The nodes and links represent cited references and cocitation relationships, respectively, from the collected articles. The reference number of the article is relative to the thickness of a node. The citation frequency at different times is reflected in the different circle colors and thicknesses of the node. The colors of links correspond directly to time slices. Nodes with high betweenness are generally thought of as pivotal points that offer crucial linkages bridging two areas of research. Table 3 presents the top 10 cited references in HIV PrEP. The articles written by Mccormack S were quoted 537 times and were the top-ranked item by citation count, followed by Baeten JM; and Thigpen MC; Cohen MS; Molina JM. High betweenness nodes can be viewed as turning points offering crucial links that bridge two fields. The top 10 cited references are ranked by betweenness centrality in Table 3, and Grant RM’s paper comes out on top, followed by Baeten JM and Molina JM.

**Table 3:** The top 10 co-cited references.

Cited frequency	Centrality	Cited reference	Source	Volume	Page
537	0	Mccormack et al. (2016)	LANCET	V387	P53
476	0.16	Baeten et al. (2012)	NEW ENGL J MED	V14	P399
400	0.13	Thigpen et al. (2012)	NEW ENGL J MED	V329	P423
400	0.02	Cohen et al. (2016)	NEW ENGL J MED	V61	P830
389	0.09	Molina et al. (2015)	NEW ENGL J MED	V321	P2237
359	0.2	Grant et al. (2010)	NEW ENGL J MED	V214	P2587
318	0.01	Damme and Szpir (2012)	NEW ENGL J MED	V375	P411
314	0.01	Choopanya et al. (2013)	LANCET	V63	P2083
307	0.01	Marrazzo et al. (2015)	NEW ENGL J MED	V29	P509
273	0	Grant et al. (2014)	LANCET INFECT DIS	V319	P820

**Figure 6:**
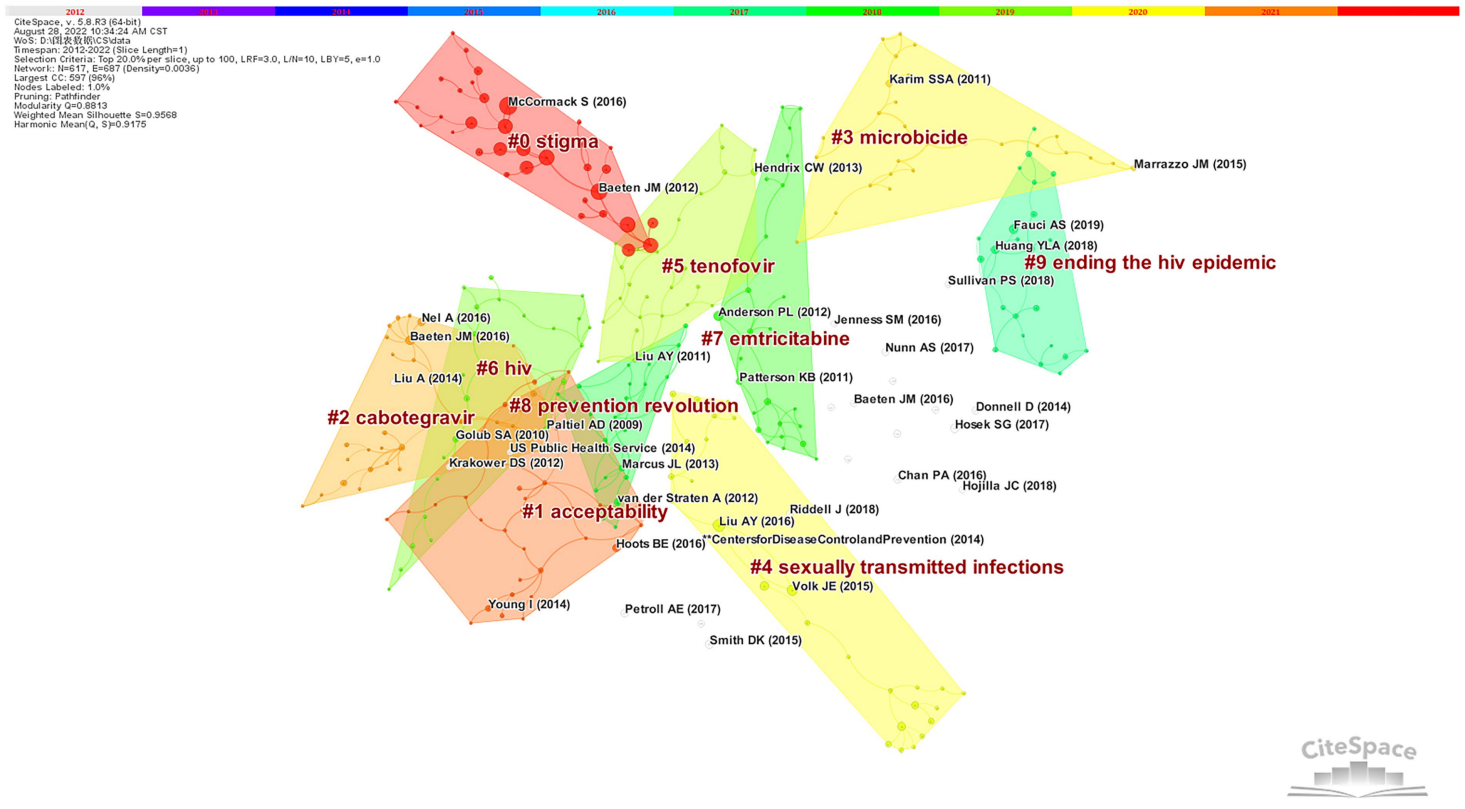
Cluster visualization based on the reference cocitation network.

The modularity Q and the mean silhouette are both used to evaluate clusters. If Q > 0.3, the network is significant, and if the silhouette >0.5, the clustering result is rational. Cocitation analysis of HIV PrEP studies produced 44 clusters, and the top 10 remained in the map (Figure 6) and were identified by their own citation terms. The findings indicated that the network had a modularity Q score of 0.8813, above 0.5, which indicates that it was reasonably decentralized into loosely coupled clusters. All silhouette scores are above 0.9, indicating acceptable homogeneity. The largest cluster, #0, was marked as “stigma” and consisted of 35 members (2015). This is followed by “cabotegravir” (#2) and “microbicide” (#3), which have 32 and 28 members, respectively. The fourth is Cluster ID #4, with the tag “sexually transmitted infections” and 28 members. The cocitation cluster markers of documents show that scholars used different technical means for studying the treatments, improvements, and promotion of HIV PrEP.

### 3.6. Main research hot spots in HIV PrEP

Keyword analysis can identify popular research areas as well as changes in relevant research hotspots and frontiers. CiteSpace will be used to analyze keywords and make the co-occurrence graph, the cluster map, the timeline view, and keywords with citation bursts.

#### 3.6.1. Keyword co-occurrence analysis

**Figure 7:**
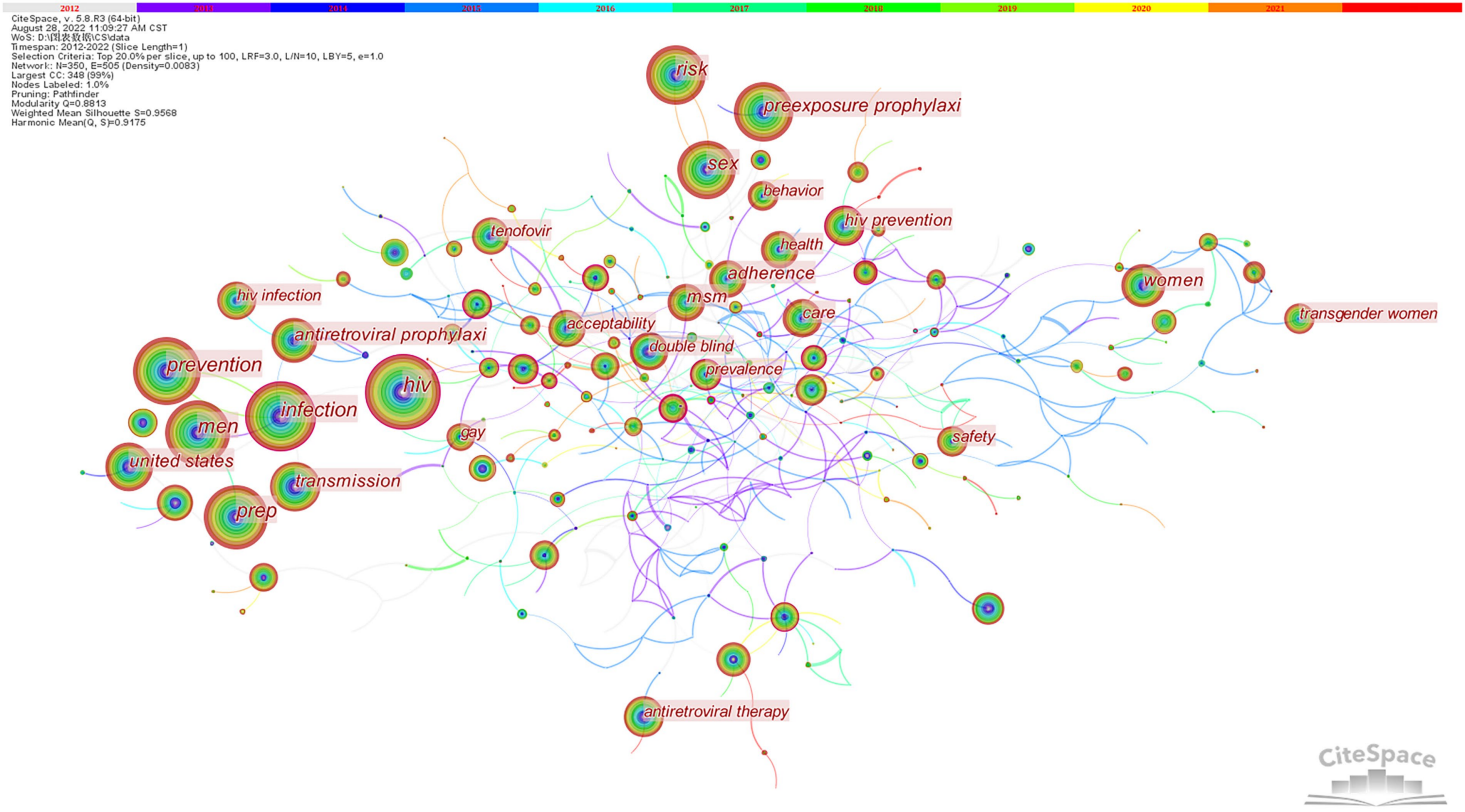
The network map of keywords.

After deleting the retrieval term and making some adjustments, Figure 7 was produced. Each node represents a keyword, and the size of each node corresponds to the co-occurring frequencies. The color of the link represents the chronological order: the oldest in gray and the latest in red. High-frequency keywords represent hot topics in a field. As illustrated in Table 4, the top five high-frequency keywords were infection (848); prevention (835); men (804); sex (579); and risk (559). Nodes marked with purple circles represent good betweenness centrality. High centrality keywords indicate the status and impact of the corresponding research content. Among these keywords, “high risk” had the highest centrality of 0.31. In addition, the centrality of vaginal transmission (0.24) and intravaginal ring (0.19) was also high. There is no doubt that controlling HIV transmission has always been a concern. Furthermore, the keywords “vaginal transmission” and “intravaginal ring” illustrated a main mode of HIV infection, which implied that the number of women in the HIV-infected population had increased.

**Table 4:** The top 10 high-centrality keywords.

Rank	Keywords	Count	Centrality
1	Infection	848	0.13
2	prevention	835	0
3	Men	804	0.01
4	Sex	579	0.01
5	Risk	559	0
6	Transmission	363	0
7	United states	362	0.06
8	Antiretroviral prophylaxi	338	0.04
9	Women	295	0
10	MSM	286	0.1

#### 3.6.2. Cluster map analysis

**Figure 8:**
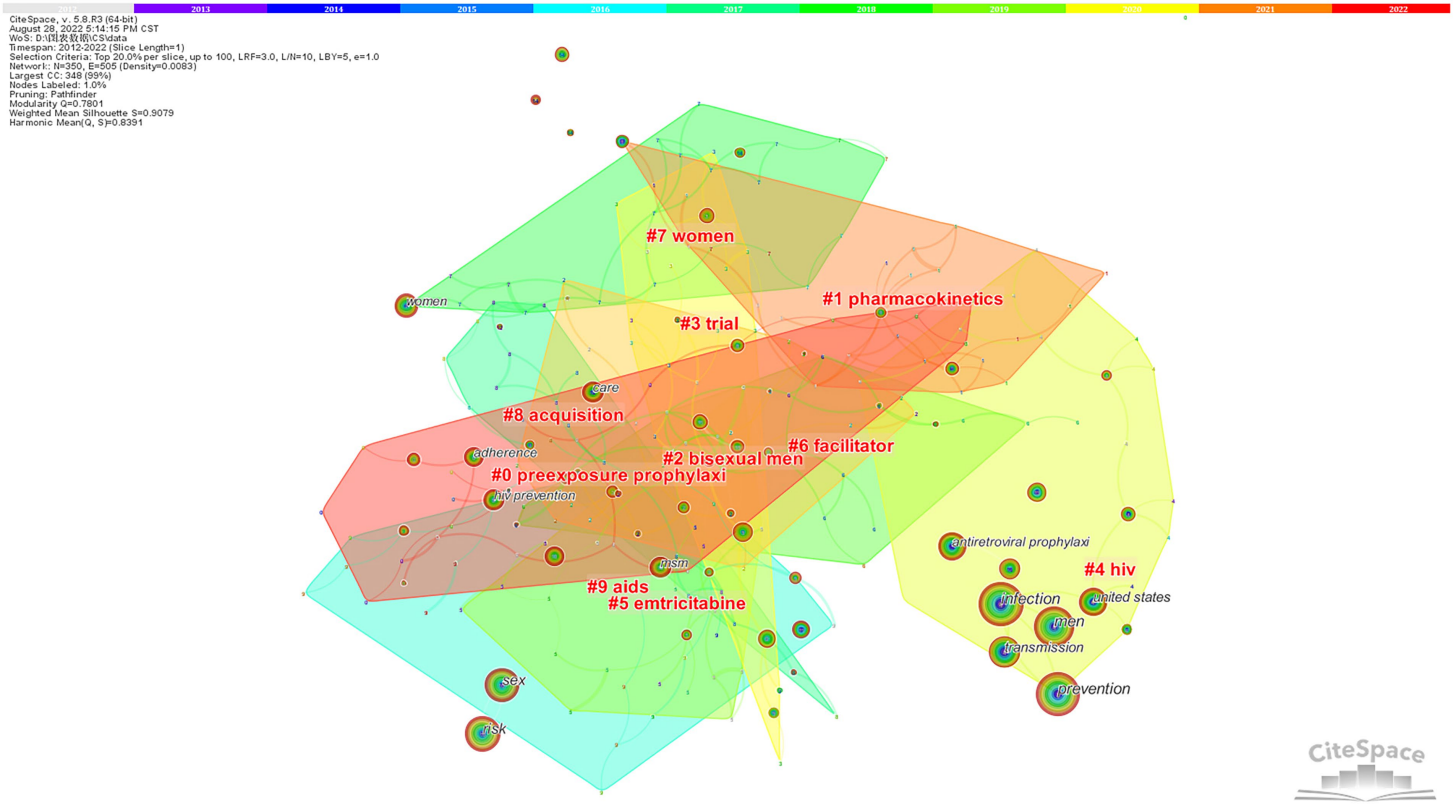
Keyword cluster analysis co-occurrence map.

We performed a cluster analysis of co-occurrence keywords that revealed the main themes. CiteSpace can determine the nature of clustering by extracting noun phrases from titles citing clusterings based on three special indicators (inverted document frequency, log-likelihood ratio, and mutual information). The log-likelihood ratio usually provides the best results. The mean profile is used to evaluate clusters. In general, a profile value of more than 0.5 means that the cluster is generally considered reasonable; if it is over 0.7, the cluster is efficient and convincing. Eventually, we obtained 19 clusters and retained the top 10 clusters after screening; the results are presented in Figure 8. Their silhouette values were all above 0.8, indicating that the results were credible (Table 5). Clustered color patches had a large overlap, indicating that different parts are closely related and influence each other. This chart illustrates the following four points: (1) Clusters # 2 and # 7 formed high-risk groups for AIDS and a variety of risk behaviors. Cluster #0 raised related issues and strategies. (2) The tag of Cluster # 1 is a necessary step in designing and optimizing the dosing regimen for clinical studies. This offers the basis for producing, determining, or clarifying a drug’s efficacy or toxicity. It can also be applied to obtain drug efficacy and toxic target organ information. (3) The randomized controlled trial is a major method mentioned in Cluster 3. Double blinding is a commonly used experimental method to optimize PrEP, which is more rigorous on the principle that neither the experimenter nor the participant is aware of which participants are in the control or experimental groups. It is widely used because it prevents the results from being influenced by the placebo effect or observer preference. (4) The nodes in Cluster #5 represent mainly nucleoside reverse transcriptase inhibitors as the first-line antiretroviral drugs for HIV PrEP, experimental animal models, and a method for the detection of the serum: the dry blood spot test.

**Table 5:** Keyword cluster analysis.

Cluster ID	Size	Silhouette	Label (LLR)	Mean (cited year)
#0	24	0.826	Preexposure prophylaxi	2014
#1	24	0.949	Pharmacokinetics	2015
#2	23	0.929	Bisexual men	2015
#3	21	0.903	Trial	2014
#4	21	1	HIV	2013
#5	21	0.908	Emtricitabine	2013
#6	21	0.936	Facilitator	2014
#7	20	0.935	Women	2015
#8	20	0.895	Acquisition	2015
#9	19	0.887	Aids	2017

#### 3.6.3. Timeline analysis

**Figure 9:**
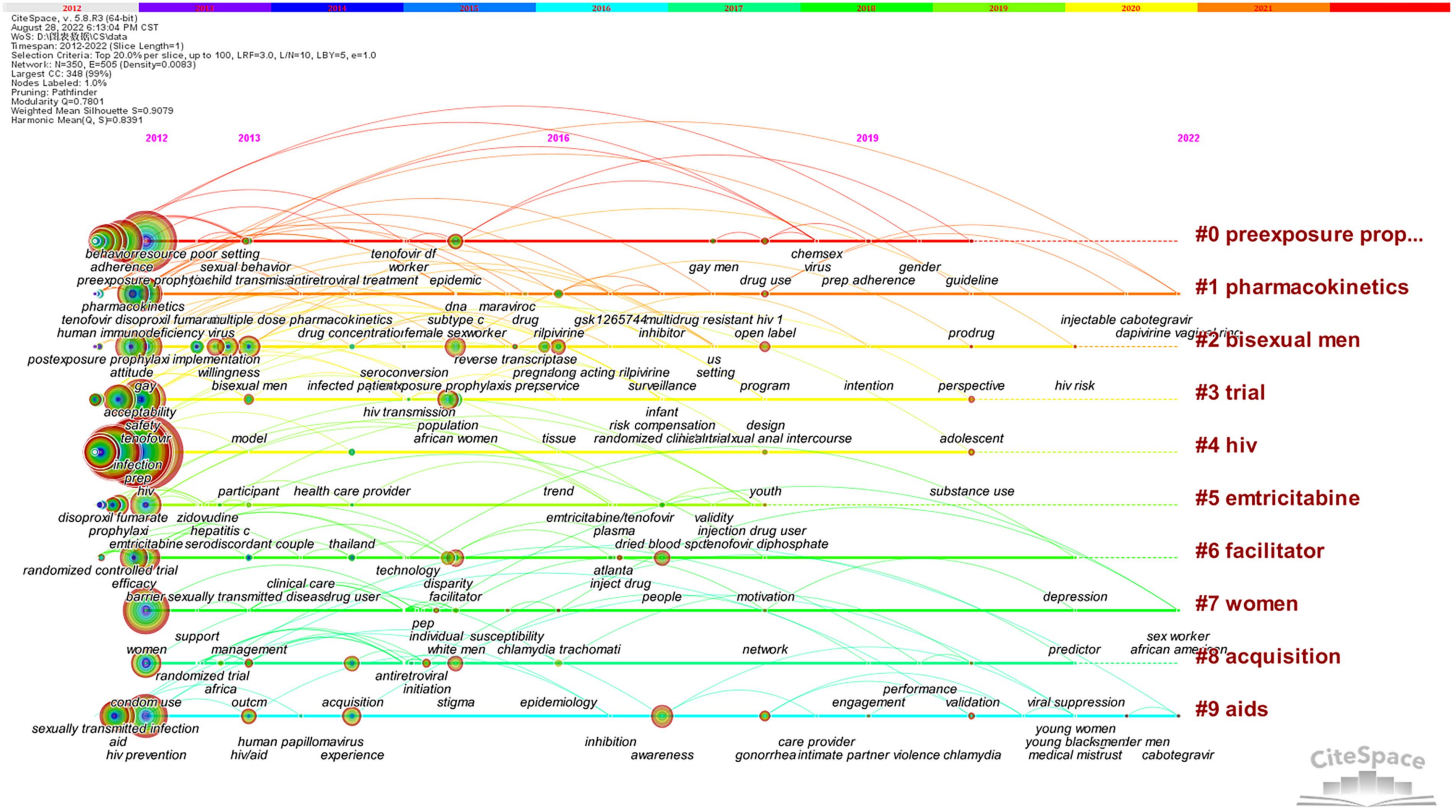
Timeline view of the keyword co-occurrence map.

To further investigate dynamic change characteristics, a timeline view analysis can show the time evolution of different cluster keywords. The historical process of HIV PrEP research is shown in Figure 9. Cluster 5 had not appeared as new nodes for a long time; thus, we can infer that experts have a basically comprehensive understanding of emtricitabine, and it is still difficult to generate new breakthroughs. Over time, Cluster 1 continued to have new keywords, and the connections between nodes indicated the importance of pharmacokinetics. The discovery, development, and updating of such drugs remain challenges and opportunities for HIV prevention.

#### 3.6.4. Keywords with citation bursts

**Figure 10:**
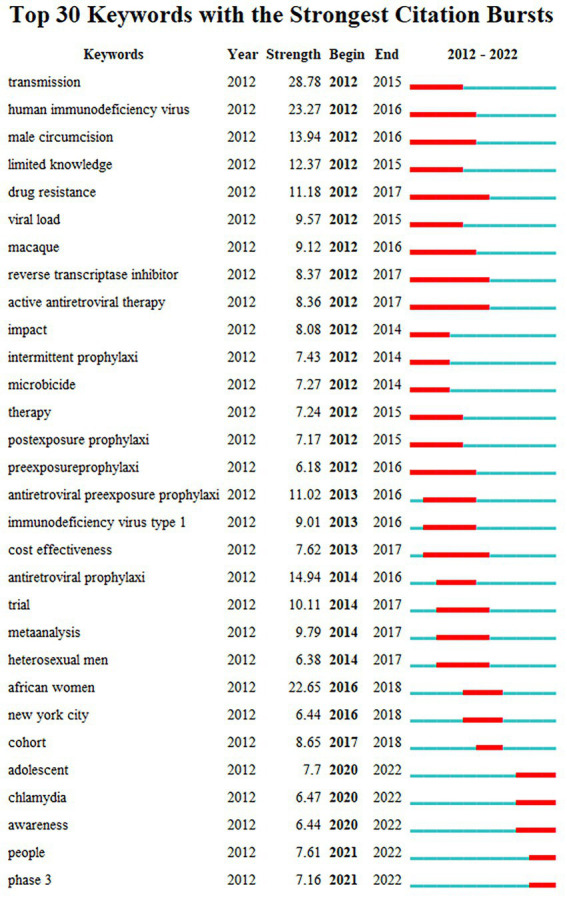
The top 30 references with the strongest citation bursts.

Burst detection algorithms are used to discover the latest trends in a field. Burst words are determined based on the growth rate of the frequency of occurrence of keywords, that is, keywords that suddenly increased in the number of citations or occurrences in a certain period of time. To explore what keywords have attracted much more attention in the academic community recently, we conducted further analysis and found a total of 51 burst keywords. The red line indicates a time period with a keyword burst, while the blue line represents the time interval. The hot frontiers of HIV PrEP in different years were analyzed according to the burst time and duration of the keywords. Figure 10 reveals the situation of the top 30 keywords with the strongest citation bursts. In terms of explosive strength, the top 5 burst keywords are “transmission” (28.78), “human immunodeficiency virus” (23.27), “male circumcision” (13.94), “limited knowledge” (12.37), and “drug resistance” (11.18). The original emergence of the topic before or since 2012 indicates that the researchers had a basic understanding of the direction of HIV PrEP a decade ago, involving viruses, treatments, drugs, epidemics, and other aspects of research. The most recent citation bursts for the keyword emerged in 2021 (phase 3, people). There are some keywords that appeared from 2013 to 2017. According to the length of a period with a keyword burst, we noticed “cost-effectiveness.” Some keywords (adolescent, awareness, chlamydia, phase 3, people) with citation bursts persisted until 2022; these words cover the frontier of the current research topic.

## 4. Discussion

In light of the fact that PrEP is a very promising tool for HIV prevention, it has attracted the attention of scholars around the globe as a significant research object. HIV PrEP has undergone rapid scientific development over the past decade, and its potential to slow the epidemic continues to be explored. To improve and innovate HIV PrEP, analyses to better understand the evolution and orientation of this research are pivotal for researchers. Covering the vast majority of related articles from the WoSCC, this paper presents both visual and scientometric analyses of HIV PrEP. Based on 3,243 documents, research network patterns and hotpots of HIV PrEP research were generated from 2012 to 2022 (October 31).

### 4.1. General information

The first paper about HIV PrEP in the WoSCC, entitled “Potential Effect of HIV Type 1 Antiretroviral and Herpes Simplex Virus Type 2 Antiviral Therapy on Transmission and Acquisition of HIV Type 1 Infection,” was published in the Journal of Infectious Diseases by Connie L. Celum, Noah J. Robinson, and Myron S. Cohen (Celum et al., 2005). It is the prototype of the HIV PrEP research field. In the beginning, some authors of which expressed a pessimistic view of the effectiveness and prospects of this approach, and the relevant research literature is scarce (Reuter et al., 2009). In 2010, a clinical trial of HIV-seronegative men or transgender women who had sex with men (iPrEx) showed that after adjustment for medication adherence in using emtricitabine and tenofovir disoproxil fumarate (FTC–TDF), the risk of HIV infection was reduced by 92% in subjects with detectable plasma drug levels (Grant et al., 2010). In 2011, another clinical study showed that in heterosexual men and women who had a known HIV-1-infected partner, daily intake of FTC/TDF reduced the risk of acquiring HIV-1 by 90% with detectable tenofovir (Baeten et al., 2012). These two trials demonstrated that PrEP is extremely effective in preventing HIV infection. After that, increasing attention was paid to HIV PrEP. Although it is unclear what the statistics will be for 2022, there were already 395 articles on October 31. We speculate that the total number of documents will climb after 2021. The results indicate that research on HIV PrEP will continue to increase rapidly, illustrating scholars’ persistent interest in HIV PrEP.

The top 10 authors engaged in the study of HIV PrEP contributed 765 articles, accounting for 23.59% of the total number of publications. Prolific authors collaborated closely. Researchers interested in the area should pay greater attention to the publications of high-influence authors since they tend to be more thoroughly explored in the field. This will help researchers better comprehend the research in the field. Among them, Jared Baeten and Connie Celum led a study called “Partners PrEP” in 2011, a pre-exposure prevention study for heterosexual couples in Kenya and Uganda. This confirmed that high-risk individuals are much less likely to become infected while taking daily HIV treatment pills, which greatly contributed to the development of HIV PrEP medicine and helped launch the first AIDS prevention drug.

The publications on HIV PrEP research originated primarily in the USA (70%). Therefore, we suggest that related studies could be conducted in other regions to obtain more comprehensive findings. The USA also has the second-highest central value and plays a vital intermediary role in facilitating the integration and exchange of HIV PrEP research achievements internationally.

Frequently cited studies have a great influence on their respective fields. The articles written by Mccormack S were quoted 537 times and were the top-ranked item by citation count. The study proved that tenofovir-emtricitabine is an effective oral PrEP and that risk compensation cannot offset its benefit. It dispelled fears that the effectiveness of this treatment would be reduced in a real-world setting (McCormack et al., 2016). Another article, considering individual differences in HIV infection risks, suggested that further assessment is needed to determine the best means of promoting and delivering PrEP to different populations. There is also a need to take into account diverse behaviors and tailor regimens to meet the needs of each individual (Dunn et al., 2016).

By analyzing the literature with a high co-citation frequency, the knowledge foundation of the subject could be obtained. Grant RM’s paper is at the top. Researchers concluded that oral FTC-TDF protects against HIV infection among subjects, and detectable blood levels are strongly correlated with protection. In particular, among subjects with unprotected receptive anal intercourse, which is the main mode of HIV transmission, efficacy was increased (Grant et al., 2010). Previous reports confirmed that heterosexual men were partially protected by male circumcision and that tenofovir 1% vaginal gel partially protected heterosexual women. Nevertheless, these methods do not protect those exposed to rectal mucosa (Auvert et al., 2005; Vibholm et al., 2012). The paper written by Grant RM was the first to demonstrate the safety and utility of tenofovir in unprotected receptive anal intercourse individuals. The second paper was written by Baeten JM, who reported that oral TDF and FTC/TDF provide significant protection for heterosexual men and women against HIV-1 infection. As mentioned above, these first two articles drove the use of clinical PrEP. The third paper, written by Molina et al. (2015), also supported the conclusion that taking TDF-FTC daily prevented HIV infection in heterosexual adults who were sexually active. Furthermore, it mentioned that the effects of prophylactic daily TDF–FTC therapy remain unknown in terms of long-term safety, including its effects on bone mineral density (Thigpen et al., 2012). Follow-up studies also examined PrEP’s safety and indicated that although the glomerular filtration rate and bone density are at risk of reversible declines, PrEP significantly hampers HIV infection at the population and broad individual level, which outweighs this risk (Mugwanya and Baeten, 2016). Future research should focus on reducing these risks and other adverse effects to improve safety and adherence to PrEP.

In reference co-citation cluster analysis, the largest cluster, “stigma,” specifically contained rejection by partners, stereotypical portrayals of promiscuity and chemsex, labeling of both drug and user, and so on (Dubov et al., 2018). HIV prevention efforts have been stalled by these potential barriers. Throughout the LGBTQ+ community, healthcare system, and society as a whole, HIV/AIDS stigma has had an insidious impact. Despite the initial resistance to PrEP abating over time, those at greatest risk are still impeded from receiving effective preventive care because of stigmatized views of PrEP (Herron, 2020). Removing the stigma of HIV PrEP, changing people’s attitudes, and increasing the acceptance of PrEP still require a series of measures. The second one (#1) is “acceptability,” which has 32 member articles and an average publication year of 2012.

### 4.2. Emerging topics

The frequency and centrality of keywords show that the current international research hotspots for HIV PrEP are infection, women, and attitude. The keywords and references in the clusters reveal that the dominant focus areas of HIV PrEP research are epidemic, high-risk group and behaviors, pharmacokinetics, and acceptability of the treatment. According to the burst detection analysis, the top five burst keywords are transmission, HIV, circumcision, limited knowledge, and drug resistance. Among the emerging trends and research frontiers of the current research theme are awareness, chlamydia, and LAI PrEP, which are expected to be further studied. People, especially teenagers, are the main subjects. Through the analysis, we discover the following:

(1) Adolescent and People: The subject of the HIV PrEP study has changed from the previous macaque to the human, especially the adolescent.

(2) Chlamydia: In the world, chlamydia trachomatis (chlamydia) is the most commonly diagnosed bacterial sexually transmitted infection (STI). Research shows that HIV-infected men who have sex with men (MSM) have a high prevalence of asymptomatic rectal chlamydia (Xiridou et al., 2013). From a biological standpoint, there was evidence that chlamydia infection increased one’s susceptibility to HIV acquisition, with rectal infection being the most likely to do so (Kasaie et al., 2019). Relevant experimental conclusions prove that HIV transmission could be facilitated by chlamydia infection among MSM. Routine chlamydia screening can decrease chlamydia prevalence among HIV-infected MSM (Xiridou et al., 2013).

(3) Awareness: PrEP awareness is one of the first steps in PrEP uptake (Parsons et al., 2017; Raifman et al., 2019). PrEP awareness refers to having at least heard of PrEP but not necessarily knowing what it is or the exact guidelines for using it. In contrast, to be knowledgeable about PrEP, one must understand its effectiveness, the eligibility requirements for using it, and how to access it (Sophus and Mitchell, 2019). Prior studies have found that once people become aware of PrEP, they may seek knowledge to improve their understanding of the drug, which may increase their willingness to use it (Auerbach et al., 2015; Walters et al., 2017). Despite PrEP’s importance and usefulness as an HIV prevention strategy, the global population is not well aware of it (Sophus and Mitchell, 2019). Awareness of HIV PrEP is not equal among different races or ethnicities. As previous studies have shown that awareness is important for PrEP uptake, it is imperative to explore ways to increase PrEP awareness, and further efforts are needed to address disparities in it. In addition to the experience that has been found to contribute to awareness-raising and the use of non-PrEP HIV prevention, several methods are available for increasing PrEP awareness, including mass media campaigns, e-health, mobile health, and education programs (Sophus and Mitchell, 2019). Studies on whether these methods can raise PrEP awareness and are appropriate for specific high-risk populations are still in their infancy, so more research and validation are needed.

(4) Phase 3: The search of relevant literature revealed that the term was derived from an experiment with a potential regimen of long-term injection (LAI) PrEP. These drugs offer long-term protection to people at risk of HIV through multimonthly injections, such as lenacapavir (dosed every six months). It improves the accessibility and adherence to antiretroviral drugs for treatment and prevention, lowering barriers to daily oral medication use (Sahloff et al., 2022). In recent studies, the establishment of optimal dosing strategies to maximize the effectiveness of LAI PrEP has been the focal point, and its safety and pharmacokinetic profile have been the main criteria for evaluation. The drug specifically targets cabotegravir and rilpivirine, which are major candida medications (Markowitz et al., 2017; Nyaku et al., 2017).

Based on the length of time the keyword popped up, we noted the word “cost-effective” appeared between 2013 and 2017. However, the efficacy of PrEP in preventing infection has been demonstrated. Due to its high sensitivity to the price of the medicine, the HIV risk of people taking PrEP, and their level of adherence, the cost-effectiveness of PrEP is also a controversial issue (Coleman and Prins, 2017). The groups most likely to be infected may not be able to afford long-term medication. However, a few years later, some scholars argued that the debate should not be whether PrEP is cost-effective but rather the possible impact of implementing it (Zioga et al., 2020). As a result, several strategies were adopted to address the situation, and some countries rolled out national programmes with subsidized costs or indicated that PrEP would be available free of charge to those who use it. Various routes have been opened through society to obtain PrEP, mainly *via* the internet. Where there is clinical support, such as a prescription or evidence of any other HIV-negative or renal function reports, the safety and follow-up of individuals who purchased online can be ensured. Some internet sites did not even require these proofs (Coleman and Prins, 2017).

### 4.3. Limitations

However, this study also had latent limitations. First, the data only included literature downloaded from WoSCC, which made the data not sufficiently comprehensive, and the analysis may be incomplete. Second, research is continuously updated in the database. With future breakthroughs in HIV PrEP research, the number of related papers may grow rapidly. Therefore, there may be discrepancies between bibliometric analysis data and actual research.

## 5. Conclusion

CiteSpace is a widely utilized instrument for knowledge graph analysis and can visualize research findings involving the distribution of authors, countries, journals, references, and keywords. Significant progress has been made over the past decades in the main areas of knowledge in HIV PrEP research, which include factors of HIV transmission and susceptibility, drug innovations and breakthroughs, and treatment acceptability. Moreover, long-term injection PrEP, the impact of chlamydia on HIV PrEP, and individual awareness of and attitudes toward HIV PrEP are among the major ongoing research trends. The findings from our study provide professionals with an intuitive understanding of research patterns and trends, directing future studies to address public acceptance, unravel pharmaceutical mechanisms, and exploit novel approaches to the current drug development pipeline for PrEP.

## Data availability statement

The original contributions presented in the study are included in the article/Supplementary material, further inquiries can be directed to the corresponding author.

## Author contributions

YL and XC conceived and designed the study. XC analyzed the data and wrote the manuscript. Both of the authors read and approved the manuscript.

## Funding

This work was supported by the Xinglin Scholar Research Promotion Project of Chengdu University of TCM (grant number XSGG2020001).

## Conflict of interest

The authors declare that the research was conducted in the absence of any commercial or financial relationships that could be construed as a potential conflict of interest.

## Publisher’s note

All claims expressed in this article are solely those of the authors and do not necessarily represent those of their affiliated organizations, or those of the publisher, the editors and the reviewers. Any product that may be evaluated in this article, or claim that may be made by its manufacturer, is not guaranteed or endorsed by the publisher.
