# Supplementary figures and images for: Knowledge domain and emerging trends in HIV pre-exposure prophylaxis: A visualization analysis via CiteSpace
Source: Front Microbiol. 2023 Mar 16;14:1099132. doi: 10.3389/fmicb.2023.1099132 (PMC10060873; doi:10.3389/fmicb.2023.1099132)

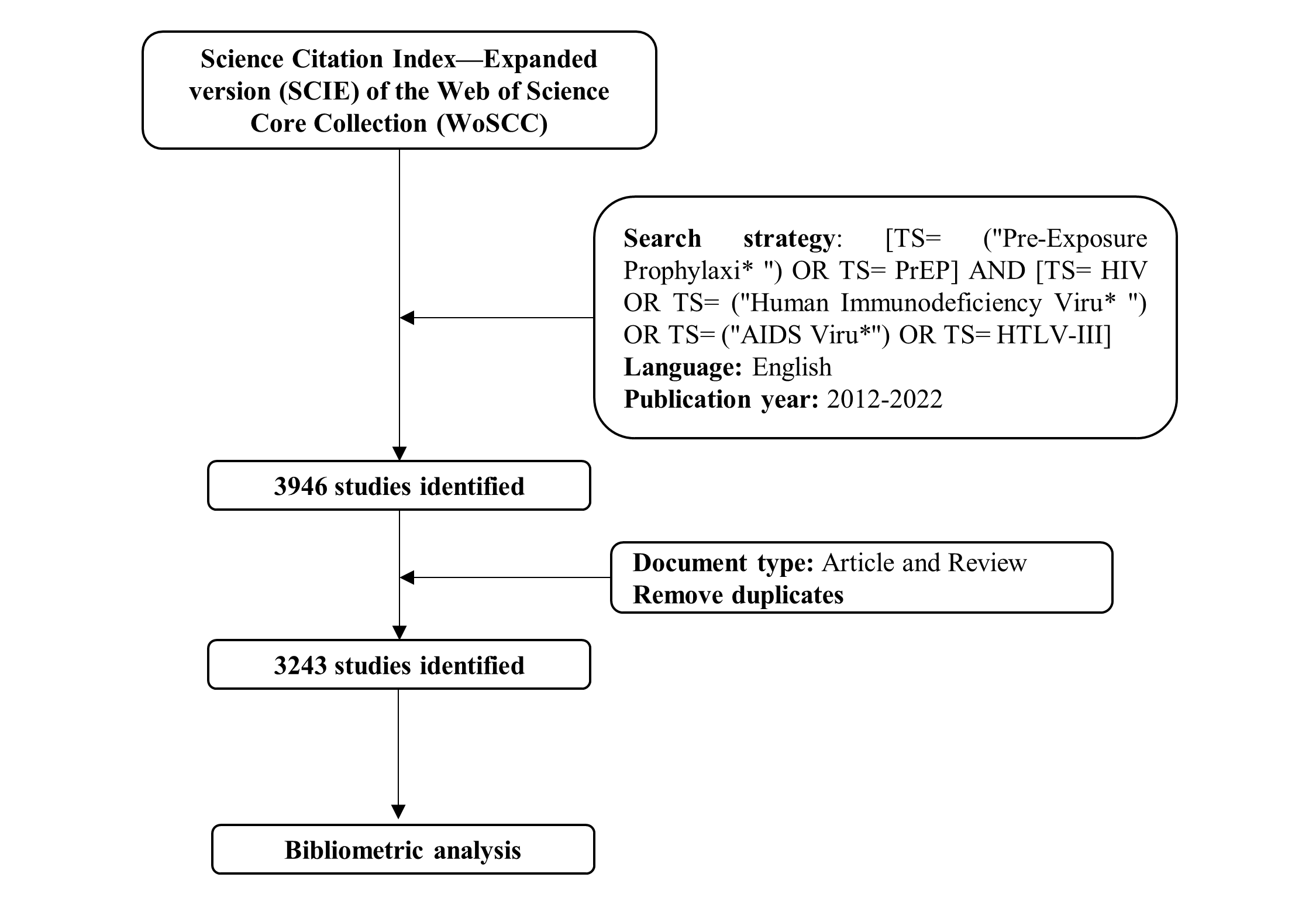

Supplement: Supplementary file 2 [file Image_1.PNG]
